# The effect of global warming on the Australian endemic orchid *Cryptostylis leptochila* and its pollinator

**DOI:** 10.1371/journal.pone.0280922

**Published:** 2023-01-30

**Authors:** Marta Kolanowska, Ewa Michalska

**Affiliations:** Faculty of Biology and Environmental Protection, Department of Geobotany and Plant Ecology, University of Lodz, Poland; Instituto Federal de Educacao Ciencia e Tecnologia Goiano - Campus Urutai, BRAZIL

## Abstract

Ecological stability together with the suitability of abiotic conditions are crucial for long-term survival of any organism and the maintenance of biodiversity and self-sustainable ecosystems relies on species interactions. By influencing resource availability plants affect the composition of plant communities and ultimately ecosystem functioning. Plant-animal interactions are very complex and include a variety of exploitative and mutualistic relationships. One of the most important mutualistic interactions is that between plants and their pollinators. Coevolution generates clustered links between plants and their pollen vectors, but the pollination and reproductive success of plants is reduced by increase in the specialization of plant-animal interactions. One of the most specialized types of pollination is sexual deception, which occurs almost exclusively in Orchidaceae. In this form of mimicry, male insects are attracted to orchid flowers by chemical compounds that resemble insect female sex pheromones and pollinate the flowers during attempted copulations. These interactions are often species-specific with each species of orchid attracting only males of one or very few closely related species of insects. For sexually deceptive orchids the presence of a particular pollen vector is crucial for reproductive success and any reduction in pollinator availability constitutes a threat to the orchid. Because global warming is rapidly becoming the greatest threat to all organisms by re-shaping the geographical ranges of plants, animals and fungi, this paper focuses on predicting the effect of global warming on *Cryptostylis leptochila*, a terrestrial endemic in eastern Australia that is pollinated exclusively *via* pseudo copulation with *Lissopimpla excelsa*. As a species with a single pollinator this orchid is a perfect model for studies on the effect of global warming on plants and their pollen vectors. According to our predictions, global warming will cause a significant loss of suitable niches for *C*. *leptochila*. The potential range of this orchid will be 36%-75% smaller than currently and as a result the Eastern Highlands will become unsuitable for *C*. *leptochila*. On the other hand, some new niches will become available for this species in Tasmania. Simultaneously, climate change will result in a substantial expansion of niches suitable for the pollinator (44–82%). Currently ca. 71% of the geographical range of the orchid is also suitable for *L*. *excelsa*, therefore, almost 30% of the areas occupied by *C*. *leptochila* already lack the pollen vector. The predicted availability of the pollen vector increased under three of the climate change scenarios analysed. The predicted habitat loss is a serious threat to this orchid even with the potential colonization of Tasmania by this plant. In the reduced range of *C*. *leptochila* the pollen vector will also be present assuring fruit set in populations of this orchid. The genetic pool of the populations in New South Wales and Queensland will probably be lost.

## Introduction

Species interactions are crucial for maintaining natural biodiversity and are essential for preserving self-sustainable ecosystems [1, 2]. By affecting resource availability and habitat structure plants affect plant communities and ultimately ecosystem functioning [3, 4]. Plant-plant interactions and their role in mediating the effect of environmental transformation are discussed by Brooker [5]. Facilitation among plants is considered to be a significant driver of ecosystem structure and functioning, especially in regions characterized by harsh environmental conditions [6]. On the other hand, competition for resources (water, nutrient, sunlight) not only influences the composition of communities it also has a role in shaping the distribution of species, as well as their evolution [7]. Plant-fungi interactions can also be beneficial or detrimental for plants. Fungal pathogens negatively affect plant physiology, whereas mutualistic fungi may enhance host defence against pathogens and symbiotic fungi can also improve plant nutrient uptake [8]. Plant-animal interactions are very complex and include a variety of biological relationships. Among these, plant-herbivore relations are examples of exploitation, whereas mutualistic connections include plants providing nesting sites and/or food rewards to ants, which often protect plants from herbivores or competing plants. Plant-pollinator and plant-seed disperser interactions are usually beneficial for both partners. The process of pollen transfer most often involves insects, birds or bats [9]. Noteworthy, coevolution generates clustered links between plants and their pollinators or seed dispersers [10], but the stability of pollination and reproductive success of plants is reduced by increased specialization of the plant-animal interaction [11–13].

One of the most specialized types of pollination is sexual deception (so-called “pseudo copulation”) which occurs almost exclusively in Orchidaceae [14, 15]. In this Pouyannian mimicry plants lure their pollinators with fraudulent sex signals [16, 17] and the pollen transfer requires specific visual and chemical adaptations of the plant. Species utilizing sexual deception depend on a single or very few species of insects [18–23].

Australia is a centre of diversity of plants pollinated *via* sexual deception, with 11 orchid genera luring insects with false sex signals [16]. These plants are found in a variety of habitats: woodlands (27%), shrubland (16%), forests with grassy or shrubby bushes (12%), damp spots near streams (11%) and exposed rocky outcrops (10%) [24]. Most species can grow in several habitats [16, 25–31]. Unfortunately, numerous Australian orchids are listed as extinct, threatened or rare [32] and this catalogue includes numerous taxa pollinated *via* sexual deception, e.g. *Caladenia* (>80 species), *Chiloglottis* R. Br. (7 species), *Cryptostylis* R. Br. (4 species), *Drakaea* Lindl. (5 species) and *Pterostylis* R. Br. (> 40 species).

*Cryptostylis leptochila* F. Muell. *ex* Benth. the small tongue orchid is an Australian endemic. It is characterized by green leaves the under surface of which is reddish-purple. Plants produce 5–15 flowers with green, linear sepals and petals and a dark red, oblong-linear lip. The lip of this species is densely covered with glandular hairs and the callus forms a ridge, which is often discontinuous, flanked on either side by an irregular row of shiny black calli [33]. *C*. *leptochila* is only pollinated by *Lissopimpla excelsa* Costa [Pimplinae, Ichneumonidae], the so called orchid dupe wasp [34, 35]. The male wasp faces the top of the upright orchid lip and its abdomen and genital claspers probe the curved pocket at base of the lip, where the stigma and the pollinia are located [25]. This plant-insect relationship is unique in being the only known act of pollination that elicits the pollinator to ejaculate [36]. Moreover, all Australian representatives of *Cryptostylis* share *Lissopimpla excelsa* as a pollen vector [25].

Pseudo copulation between an orchid and specific species of insect needs further study because global warming [37, 38] constitutes a serious threat to global biodiversity [39–51] and ecological interactions [52–57]. Many studies indicate that frequent, prolonged droughts adversely affect the pollination and seed dispersal of plants, which are crucial for reproduction [58, 59]. Orchids are particularly at risk [60–68] because global warming adversely affects the specific symbiotic associations of these plants with fungi and their complex relationships with pollinators. Any reduction in the availability of pollen vectors are especially dangerous for sexually deceptive plants whose long-term persistence [fruit production] depends on particular species of insects.

Undeniably, suitable environmental conditions together with the stability of a series of interactions critical for plant growth, development and reproductive success are essential for orchid survival [66]. The aim of this study was to evaluate the availability of the specific pollinator of *C*. *leptochila* in various scenarios of climate change using ecological niche modelling.

## Methods

### List of localities

The databases of the localities of *Cryptostylis leptochila* and *Lissopimpla excelsa* were compiled based on information in public facilities accessed through Atlas of Living Australia [69], which includes data from Shire of Kalamunda Biodiversity Inventor, Australia’s Virtual Herbarium, Australian National Insect Collection, BowerBird, Tasmanian Museum and Art Gallery Invertebrate Collection, Questagame, Queen Victoria Museum and Art Gallery and South Australian Museum, as well as iNaturalist.org and Global Biodiversity Information Facility [70, 71].

From the total of 1442 localities for *C*. *leptochila* and 856 for *L*. *excelsa* in the repositories only records that were georeferenced with a minimum of 1 km precision were selected (S1 Annex).

To reduce sampling bias spatial thinning was done using SDMtoolbox 2.3 for ArcGIS [72, 73]. The location data were spatially filtered at 5 km^2^, 10 km^2^, 15 km^2^, 20 km^2^ and 25 km^2^, in areas of high, medium-high, medium, medium-low and low habitat heterogeneity, respectively. This method of graduated filtering was used to maximize the number of spatially independent localities. The final database included 210 records for *C*. *leptochila* and 161 for *L*. *excelsa* (S2 Annex).

### Climatic niche modelling

Species distribution and ecological niche modelling are methods commonly used to predict the potential distribution of suitable habitats for various organisms [59, 74–78]. The modelling of the current and future distribution of the species studied was done using the maximum entropy method implemented in MaxEnt version 3.3.2 [79–81], which is based on presence-only observations. Bioclimatic variables in 30 arc-seconds of interpolated climate surface downloaded from WorldClim v. 2.1 were used for the modelling [82]. Five of 19 variables were removed from the analyses due to high correlation between them (>0.9) as indicated by Pearsons’ correlation coefficient computed using SDMtoolbox 2.3 for ArcGIS [72, 73] in order to avoid problems associated with auto-correlation. The final list of bioclimatic variables used in the analyses is provided in S1 Table. Because some previous studies [83] suggest that modelling for a small area is more reliable than calculating habitat suitability at a global scale, the area of the analysis was restricted to 5.454–44.838°S and 111.079° -156.921°E.

Predictions of the future extent of the climatic niches of *C*. *leptochila* and its pollinator in 2080–2100 were made using climate projections developed by the CNRM/CERFACS modelling group for Coupled Model Intercomparison Project (CNRM-CM6-1) for four Shared Socio-economic Pathways (SSPs): 1–2.6, 2–4.5, 3–7.0 and 5–8.5 [84–86]. The layers in 2.5 arc-minutes were re-scaled to fit bioclimatic variables. SSPs are trajectories adopted by the Intergovernmental Panel on Climate Change (IPCC), which offer a broader view of a “business as usual” world without future climate policy, with global warming in 2100 ranging from a low of 3.1°C to a high of 5.1°C above pre-industrial levels.

Because projections of future changes in the distribution and coverage of suitable niches of the orchid studied is based exclusively on bioclimatic data, the importance of other factors such as soil properties and land cover were evaluated by creating present-time models using all available variables. The models created for present time based on bioclimatic data only and based on combined bioclimatic, soil and land cover data were then compared with the known occurrences of *C*. *leptochila*. The data on the distribution of soil classes, soil pH, clay content, sand content, nitrogen content and soil organic carbon content were obtained from Global Soil Information [87] (http://www.soilgrids.org) with a 250 m^2^ resolution and upscaled to fit the resolution and extent of the bioclimatic variables. The Global Land Cover by National Mapping Organizations ver. 3 (GLCNMO) was the source of information for the land cover analyses. The complete list of variables used in the present time analyses is presented in S1 Table.

In all analyses the maximum number of iterations was set to 10000 and convergence threshold to 0.00001. The neutral (= 1) regularization multiplier value and auto features were used. All samples were added to the background. The “random seed" option provided a random test partition and background subset for each run and 30% of the samples were used as test points. The run was performed as a bootstrap with 100 replicates. The output was set to logistic. In addition, the “fade by clamping” function in MaxEnt precluded extrapolations outside the environmental range of the training data [88]. All analyses of GIS data were carried out using ArcGis 10.6 (Esri, Redlands, CA, USA). The evaluation of the created models was done using the area under the curve (AUC) [89, 90] and True Skill Statistic (TSS) [91, 92].

SDMtoolbox 2.3 for ArcGIS [72, 73] was used to visualize changes in the distribution of suitable niches of the orchid studied and its pollinator due to global warming. To compare the distribution model created for current climatic conditions with future predictions all SDMs were converted into binary rasters and projected using the Goode homolosine as a projection. The presence threshold was estimated based on the values for grids in which the species studied occur in models developed using current data. About 80% of known localities for *C*. *leptochila* are located in grids with values > 0.4 and 78% of those for *L*. *excelsa* in grids with values > 0.3. These two thresholds were used to create binary rasters [93]. To calculate the availability of the pollinator for the orchid studied the overlap in the binary models of both organisms was calculated.

## Results

### Model evaluation and limiting factors

The scores of the values of both, TSS and AUC tests were high with 0.780–0.976 and 0.965–0.990, respectively (S2 Table), which indicates the high accuracy of the models.

**Fig 1:**
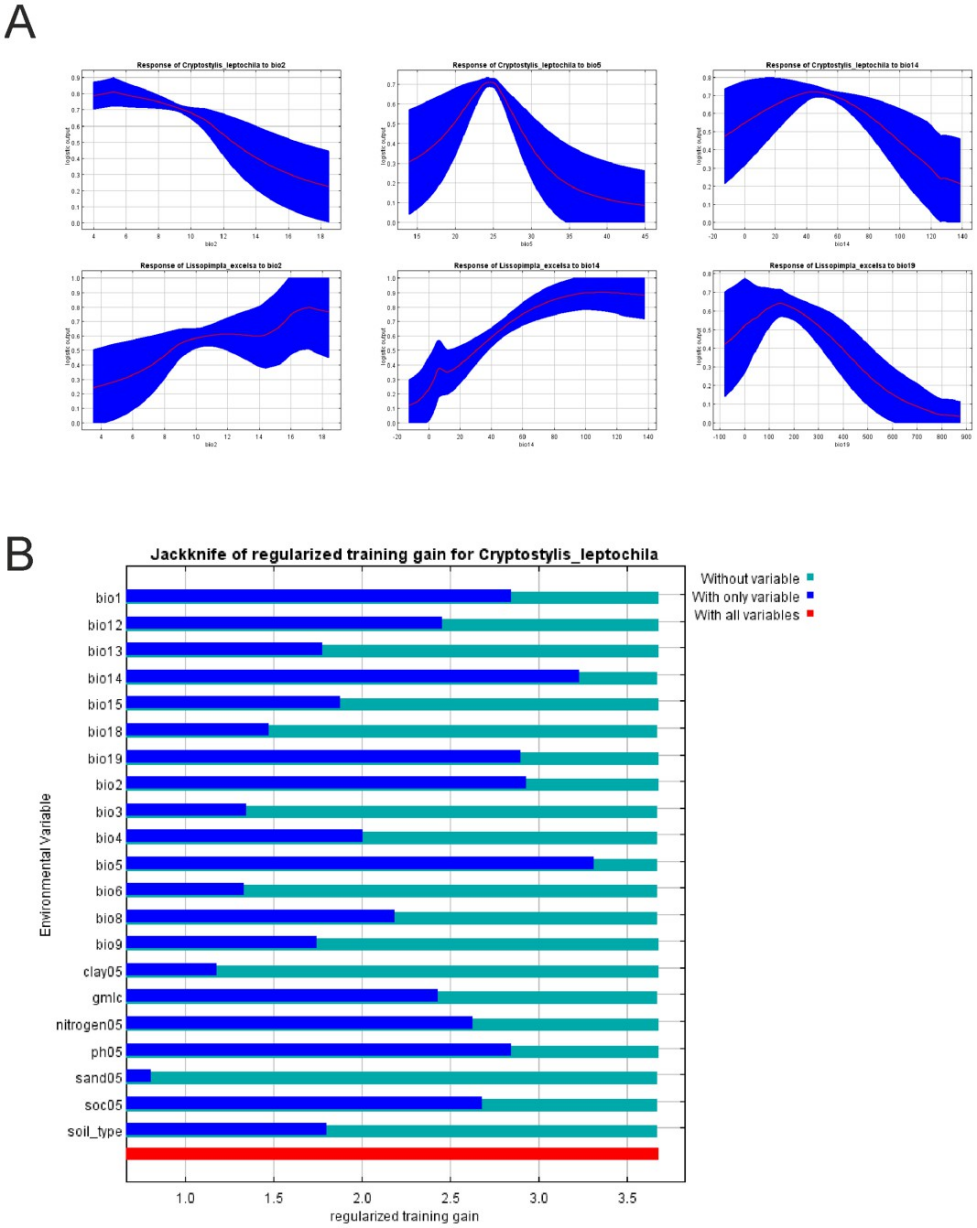
A. Effect of environmental variables on the MaxEnt prediction based only on bioclimatic data. The curves show how the predicted probability of occurrence changes as each environmental variable is varied, keeping all other environmental variables at their average value. The curves show the mean response of the 100 replicate Maxent runs (red) and the mean +/- one standard deviation (blue). B. Results of the jack-knife test of variable importance for the model created for *C*. *leptochila* using all of the variables. Values shown are averages for replicate runs.

**Fig 2:**
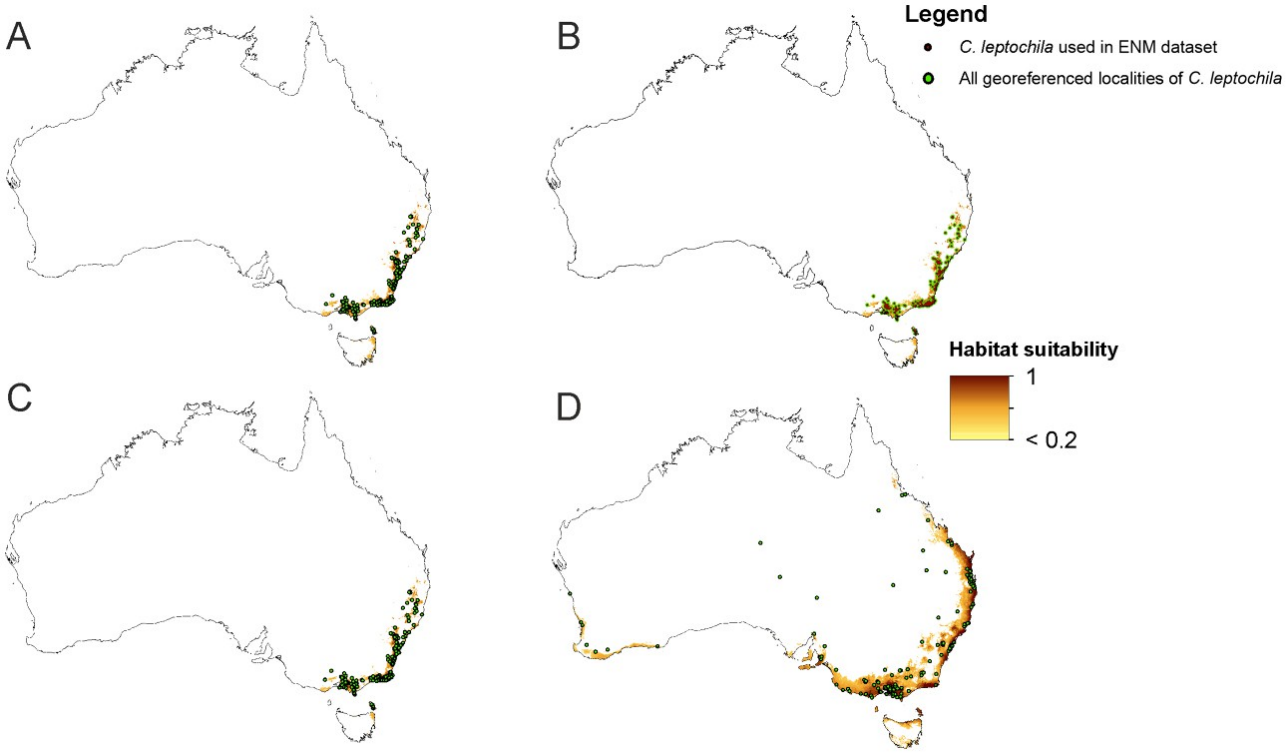
Current potential distribution of *Cryptostylis leptochila* (A-C) and *Lissopimpla excelsa* (D). A—model based on all variables and all georeferenced records of *C*. *leptochila*, B—map showing the localities of *C*. *leptochila* used in ENM studies, C-D—model based only on bioclimatic data with all georeferenced records for the species studied. Maps generated in ArcGIS based on niche modelling (MaxEnt) results.

The crucial factor limiting the distribution of *C*. *leptochila* in models based on bioclimatic data only was precipitation in the driest month (bio14–67.3%), much less important was the maximum temperature in the warmest month (bio5–10.6%) and the mean diurnal range (bio2–10.5%). For *L*. *excelsa* essential variables were the precipitation in coldest quarter (bio19–37.4%) and precipitation in the driest month (bio14–25.7%). A somewhat smaller contribution was recorded for the mean diurnal range (bio2–12.0%). The effect of environmental variables on the MaxEnt prediction is presented in Fig 1. Models created for the orchid studied based on all available variables and produced using only bioclimatic data are very similar, with a slight overestimation of the potential range predicted by the model including all variables (Fig 2). Also, the jackknife test of variable importance indicated that bioclimatic data are crucial for constructing models (Fig 1).

### Current and potential future distribution of orchid and its pollinator

**Fig 3:**
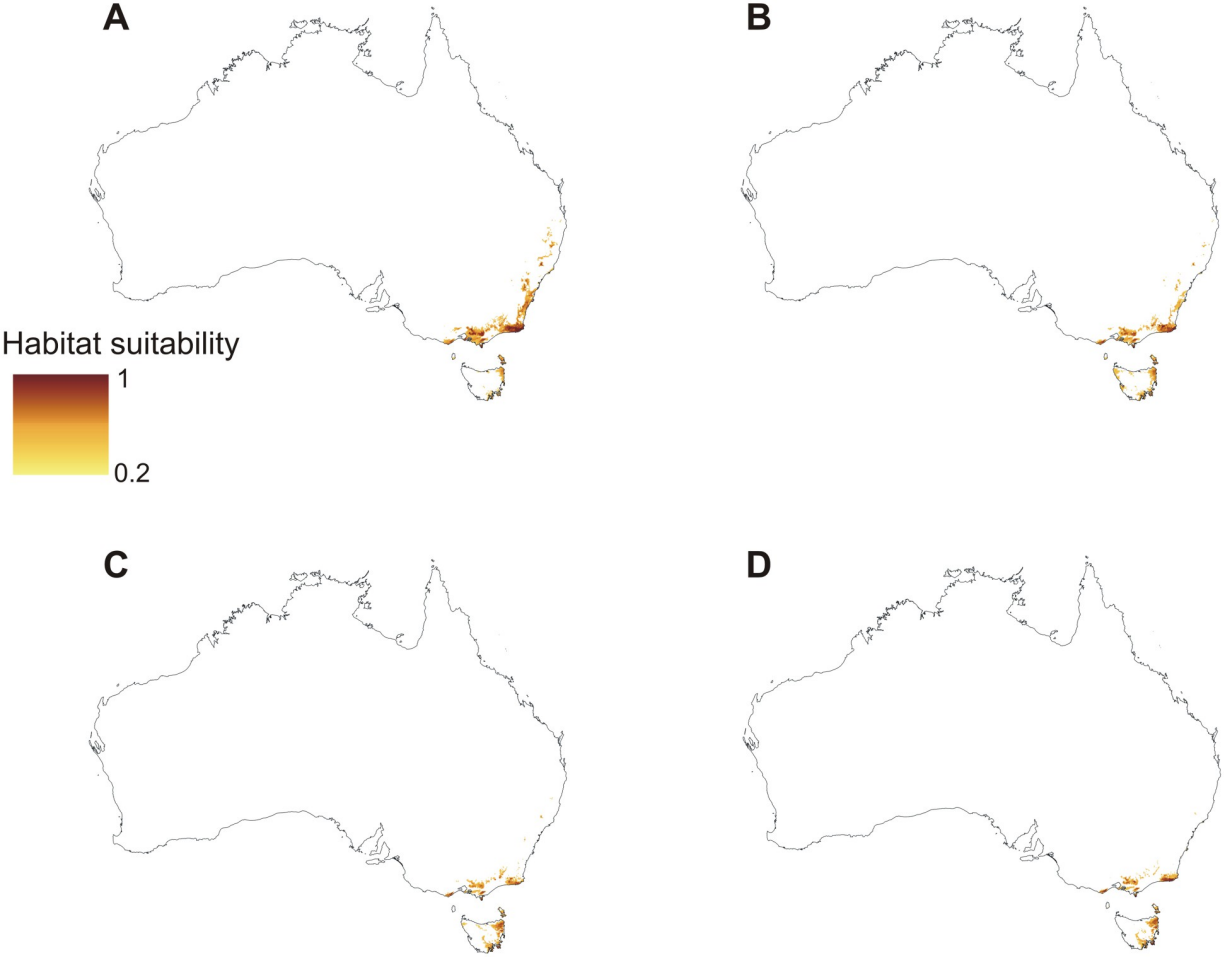
Predicted future distribution of suitable niches for *C*. *leptochila* based on the SSP1-2.6 (A), SSP2-4.5 (B), SSP3-7.0 (C) and SSP5-8.5 (D) climate change scenarios. Maps generated in ArcGIS based on niche modelling (MaxEnt) results.

**Fig 4:**
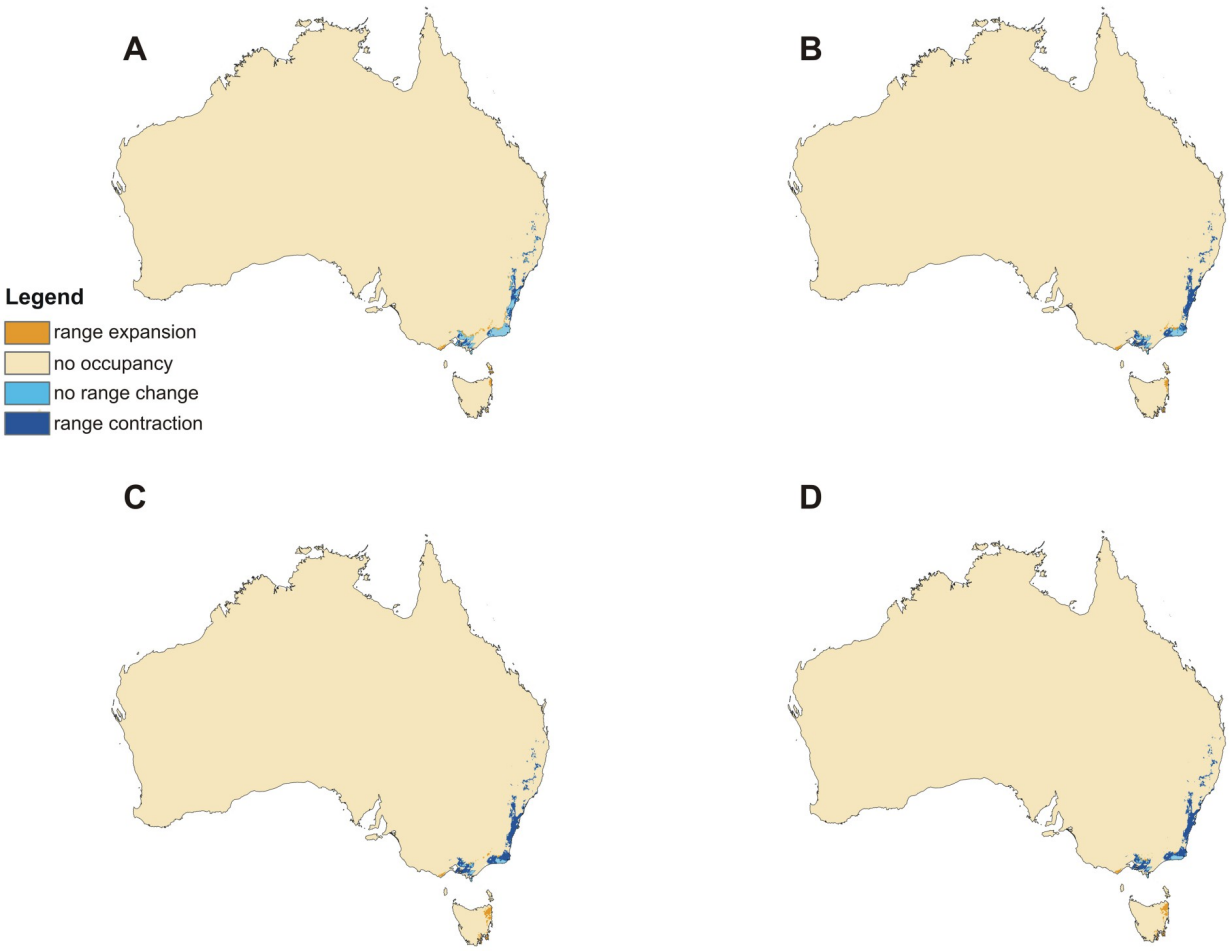
Predicted changes in the distribution of suitable niches for *Cryptostylis leptochila* based on the SSP1-2.6 (A), SSP2-4.5 (B), SSP3-7.0 (C) and SSP5-8.5 (D) climate change scenarios. Maps generated in ArcGIS based on niche modelling (MaxEnt) results.

**Fig 5:**
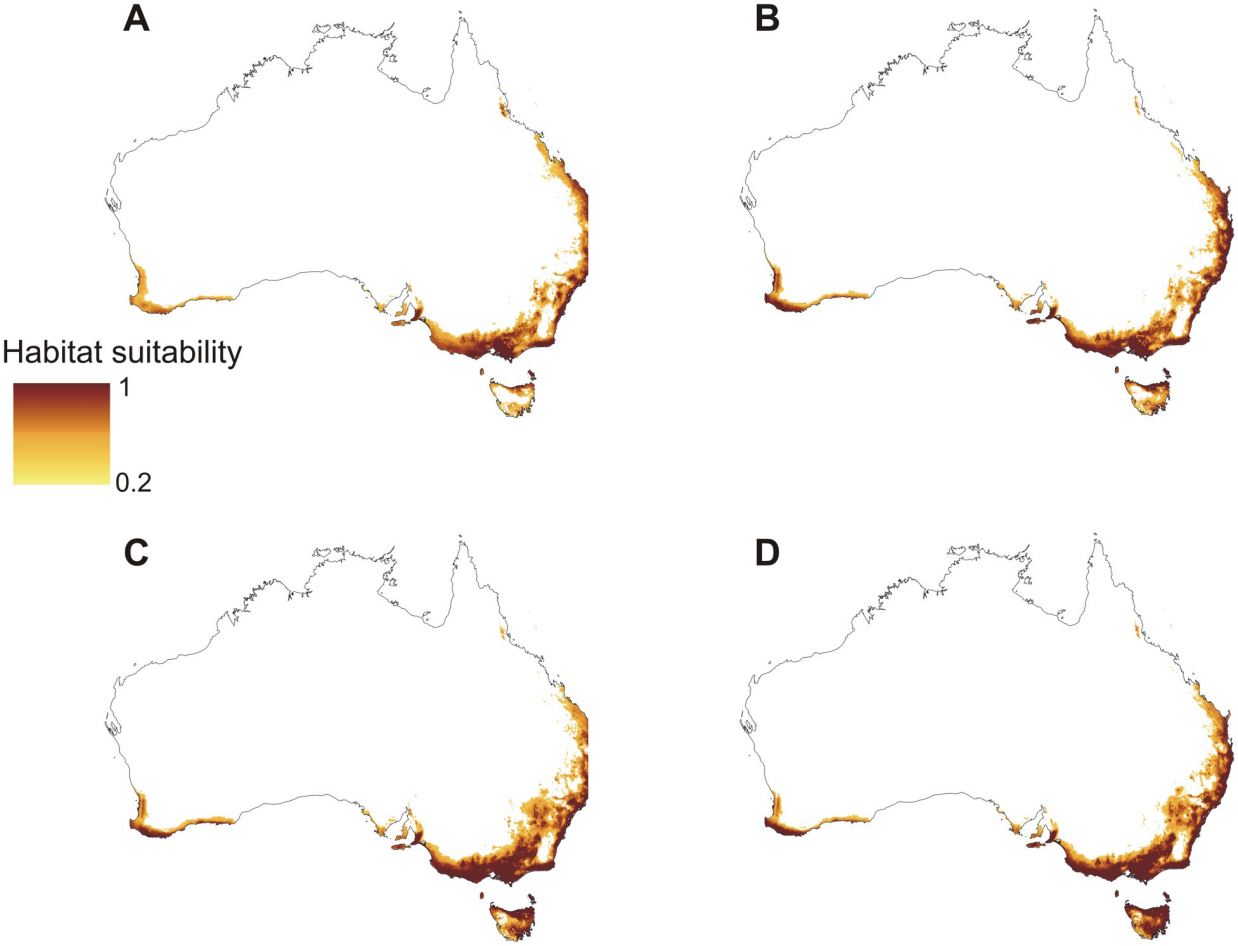
Predicted future distribution of suitable niches for *Lissopimpla excelsa* based on the SSP1-2.6 (A), SSP2-4.5 (B), SSP3-7.0 (C) and SSP5-8.5 (D) climate change scenarios. Maps generated in ArcGIS based on niche modelling (MaxEnt) results.

**Fig 6:**
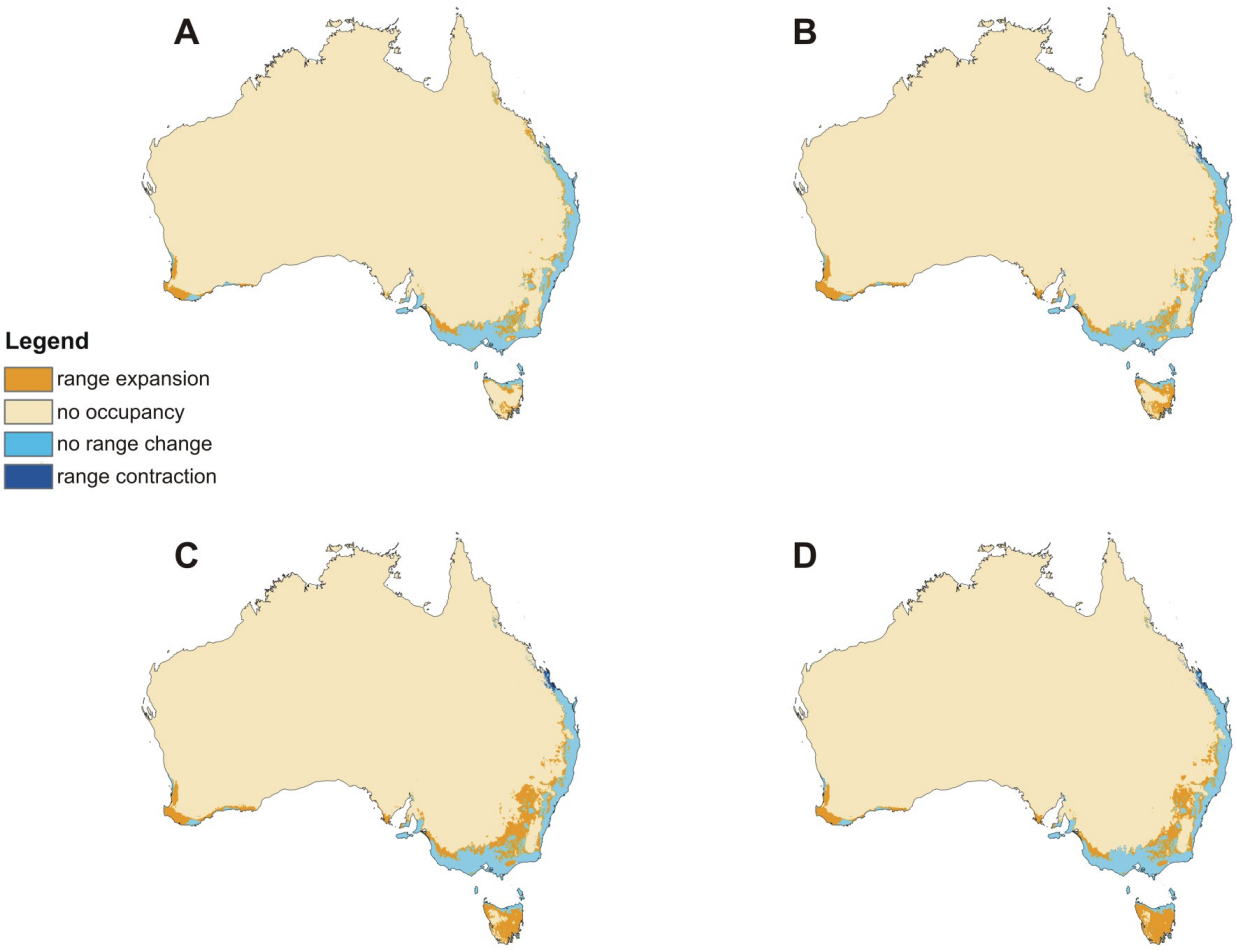
Predicted changes in the distribution of suitable niches for *Lissopimpla excelsa* based on SSP1-2.6 (A), SSP2-4.5 (B), SSP3-7.0 (C) and SSP5-8.5 (D) climate change scenarios. Maps generated in ArcGIS based on niche modelling (MaxEnt) results.

The models of current distribution of niches suitable for *C*. *leptochila* and *L*. *excelsa* are consistent with the known geographical ranges of these species (Fig 2). Based on these models global warming will result in a significant loss of suitable niches for *C*. *leptochila* (Figs 3 and 4; Table 1), but it will be favourable for *L*. *excelsa* (Figs 5 and 6, Table 1). The potential range of the orchid will be 36.064%(SSP1-2.6)-75.970% (SSP5-8.5) smaller than it is currently. In the SSP1-2.6 scenario habitat loss is expected to occur along the Eastern Highlands in New South Wales, but habitats will become available in north-east Tasmania, the Furneaux Group and some localities near Mt. Kościuszko. A similar situation is predicted by SSP2-4.5 and SSP3-7.0 models, but the habitat loss in the Eastern Highlands will be much more severe and will occur in both New South Wales and Queensland. In the SSP3-7.0 scenario, however, numerous new niches will become available for this species in southern- and northern- eastern Tasmania. In the SSP5-8.5 scenario almost all suitable niches in the Eastern Highlands will cease to exist, except for the Tasman Sea coast near the Croajingolong National Park.

**Table 1:** Changes in the coverage (km^2^) of suitable niches for *C*. *leptochila* and *L*. *excelsa*.

Species	Scenario	Range expansion	Range contraction	Overall change
*C*.*leptochila*	SSP1-2.6	7663.97	24954.10	-36.064%
*C*.*leptochila*	SSP2-4.5	6299.92	37789.28	-65.680%
*C*.*leptochila*	SSP3-7.0	7294.29	43568.92	-75.661%
*C*.*leptochila*	SSP5-8.5	6557.50	42980.17	-75.970%
*L*.*excelsa*	SSP1-2.6	135488.61	7962.62	+44.103%
*L*.*excelsa*	SSP2-4.5	155637.75	14233.45	+48.903%
*L*.*excelsa*	SSP3-7.0	254027.66	16608.12	+82.109%
*L*.*excelsa*	SSP5-8.5	220222.68	17136.10	+70.235%

On the other hand, global warming will result in a substantial increase in the coverage of niches suitable for *L*. *excelsa*. Based on SSP3-7.0, which is the most advantageous scenario for this species, the potential coverage will be 82.109% greater than currently. Based on the worst-case scenario (SSP1-2.6), the coverage of suitable niches for this species will increase to 44.103%. The habitat loss for *L*. *excelsa* predicted by all the models due to global warming is not significant and will occur near the Byfield State Forest. The increase in the coverage of suitable niches is expected to occur along the edges of the current potential range and in central and southern Tasmania, south part of Spencer Gulf and around Cape Leeuwin.

**Fig 7:**
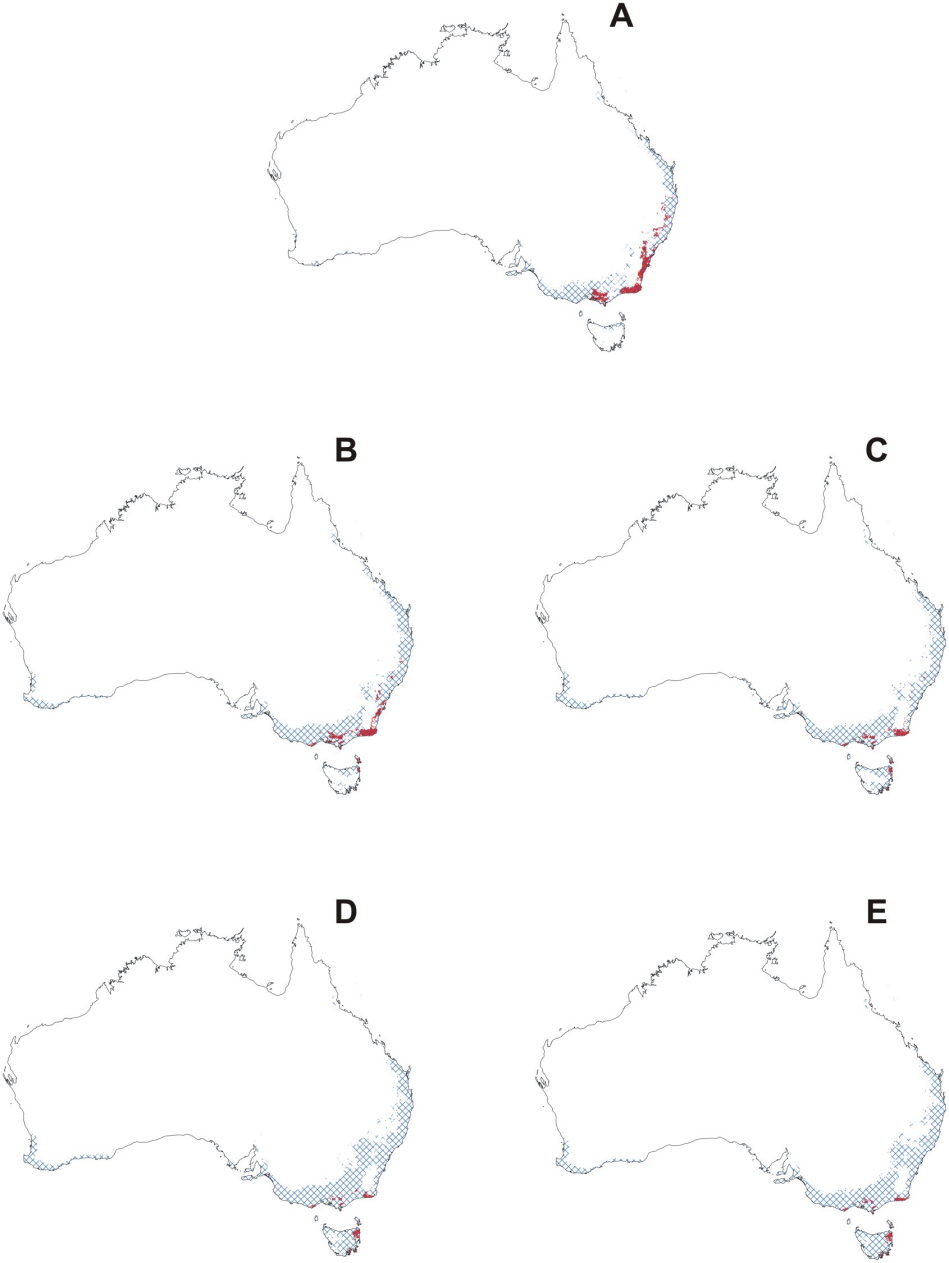
Overlap of suitable niches for *Cryptostylis leptochila* and *Lissopimpla excelsa* currently (A) and based on the SSP1-2.6 (B), SSP2-4.5 (C), SSP3-7.0 (D) and SSP5-8.5 (E) climate change scenarios. Maps generated in ArcGIS based on niche modelling (MaxEnt) results.

Currently most of the populations of this orchid are likely to be pollinated as 71.43% of this orchid’s geographical range is also suitable for *L*. *excelsa* (Fig 7). The availability of the pollen vector is predicted to increase in three climate change scenarios: SSP1-2.6 (83.33%), SSP3-7.0 (72.73%) and SSP5-8.5 (90%). In SSP2-4.5 part of the range of the orchid will overlap with the potential range of the insect.

## Discussion

The ecology of specialized pollination is very complex. In this study the possible effect of climate change on the distribution of a sexually deceptive orchid and its pollinator were explored, which revealed that the reproductive success of *C*. *leptochila* depends on many ecological interactions. The pollinator of *C*. *leptochila* is a parasitoid that is dependent on the availability of a host insects that feed on particular plants. In addition, germination of the orchid is dependent on symbiosis with mycorrhizal fungi, which are also often dependent on the occurrence of other species of plants. Here we discuss the results of our analysis and limitations of this study, which requires a more detailed insight into the ecological interactions between plants, insects and fungi.

Areas with low and very high values for precipitation in the driest month are unsuitable for *C*. *leptochila* and since global warming will significantly change the water regimes in the world it is not surprising that numerous currently suitable niches for this orchid will change and probably become unsuitable for this species. The two other variables important for the occurrence of this species are related to temperature. *Cryptostylis leptochila* prefers areas where the maximum temperature in the warmest month is ca. 25°C and the mean diurnal range is ca. 5°C. The predicted climate changes [94] indicate that Australia will be hotter, with longer and more intense heatwaves. As a result of global warming the Eastern Highlands will become unsuitable for *C*. *leptochila*, however some new niches will become available for this species in Tasmania. The predicted loss in suitable niches of 36–76% is a serious threat to the orchid even with its potential expansion in Tasmania. The genetic pool of the populations in New South Wales and Queensland will probably be lost.

In contrast to previous studies on the future of orchids pollinated *via* pseudo copulation with a single species of insect [37] this research predicts that the orchid studied will face significant habitat loss, which will be accompanied by an expansion in the geographical range of its pollen vector. There is several studies on the European sexually deceptive genus *Ophrys* L. which is currently the most diverse taxon comprising of orchids pollinated *via* pseudo copulation [95]. Hutchings *et al*. [96] as well as Robbirt *et al*. [97] studied only one of five known pollinators of *O*. *sphegodes* Mill. They provide proof of disruption of the phenology and future interactions between this orchid and a bee (*Andrena nigroaenea* Kirby) and the potential effect of climate change on another three Hymeoptera (*Andrena nitida* Müller, *Andrena ovatula* Kirby, *Andrena paucisquama* Noskiewicz) and one Diptera (*Myopa tessallatipennis* Motschulsky) that are pollen vectors for the early spider-orchid [98–101]. Tsiftis & Djordjević [102] include the presence of the exclusive pollinator of Greek *Ophrys argolica* Fleischm. *ex* Vierh. and *O*. *delphinensis* O. Danesch & E. Danesch as a variable in their models of future occurrence of these orchids. This approach, however, is misleading as the vegetative growth of bee-orchid is not dependent on the existence of a pollen vector. The methodology used by Tsiftis & Djordjević [102] is more applicable to studies on parasitic plants, which are entirely dependent on the presence of their hosts.

Our predictions indicate Tasmania as a future potentially suitable area for *C*. *leptichila* and *L*. *excelsa*. The orchid has the potential to migrate into this region as it is already present in mainland Australia as well as in Furneaux Group islands. The long-distance dispersal [> 150 km] is not uncommon in terrestrial orchids [103, 104]. Orchid dupe wasp is already present in New Zealand [105] so its migration to Tasmania is also probable.

In the limited number of niches that will still be available for *C*. *leptichila* the pollen vector will be present assuring fruit set in orchid populations. There is only one long-term study on a species of orchid pollinated *via* sexual deception [96, 97] and on one of at least five known pollinators of this taxon. This study indicates that ongoing climate warming, especially warmer springs, is likely to increase the frequency of years in which the plant experiences low pollination success. According to information in public databases over the last 70 years the flowering time of *C*. *leptochila* has not changed despite climate change. While few occurrences of *L*. *excelsa* were reported before 1980, based on the public databases records, the pollinator is available during the whole flowering period of this orchid (S3 Table). On the other hand, based on museum records Brunton Martin et al. [106] demonstrate that male orchid dupe wasps can be less abundant between May and September so that the synchronization of the flowering of the orchid and male insect activity could be threatened by climate change modifying their phenology. Interestingly, the same researchers [106] also show that populations of *L*. *excelsa* are male biased in the areas where *C*. *leptochila* occurs allowing the persistence of the pollen vector despite sperm wastage during pseudo copulation. Though global warming can potentially cause modifications in insect behaviour, the daily activity of *L*. *excelsa* on *C*. *leptochila* flowers currently does not seem to be limited by any particular climatic factor like humidity or sunlight [107, 108]. Male *L*. *excelsa* remain active over a broad range of temperatures [109] (20.1–36.8°) and based on the maps of future climatic conditions [82] the temperatures in localities where *C*. *leptochila* occurs will not exceed that tolerated by these insects.

This study explored the distribution of an orchid and its pollinator, but not the long-term survival of the orchid dupe wasp, which is also threatened by the effects of global warming. Females of *L*. *excelsa* parasitize large caterpillars such as budworms and armyworms [110] and the stability of orchid dupe wasp population depends on the availability of these larvae. For Lepidoptera one of the major threats associated with climate change are temporal mismatches with availability of food sources [111]. Global warming affects the development of insect herbivores more than their hosts leading to desynchronization of flowering time and insect activity period [112]. Increased temperatures trigger also asynchrony by supporting faster development of caterpillars than their parasitoids [111, 113]. The complex relationship of *L*. *excelsa* with the species it parasitizes could not be included in this study because the list of Lepidoptera species parasitized by orchid dupe wasp is incomplete. Also, the list of such Lepidoptera would have to be compiled and modelled to estimate their chances of survival when faced by changes in climate.

Obviously, pollinator availability is not the only ecological factor that could change due to global warming becoming a threat to *C*. *leptochila*. Orchids have numerous symbiotic relationships with endophytic fungi [114, 115] and bacteria [116]. The interaction with a mycorrhizal fungus, which provides essential nutrients for seedling development is vital for orchids [117] and the seedling recruitment success is strongly associated with the availability of a suitable fungal strain. While some orchids are mycorrhizal generalists [118], other are characterized by specific relationships with fungal genera [119]. The nature of the symbiotic relationships of *C*. *leptochila* with fungi and bacteria are poorly known and as a consequence this aspect of orchid ecology could not be evaluated in this study. In numerous mycological studies there is no species-level identification of symbiotic fungi only taxonomic units (OTUs) [120]. Without proper naming of orchid symbionts, a dataset of the distributions of particular fungi could not be created and included in the ENM analyses.

Abiotic factors are also likely to affect the distributions of orchids as germination of their seeds is dependent on soil moisture, pH and organic content [121]. Also, extreme events like heat waves, hurricanes, etc, are expected to occur more frequently as a result of climate change [122], which could substantially affect orchid populations. According to Brown [123] apart from the factors listed above, inappropriate fire regimes, groundwater extraction, spread of exotic plants and grazing should be considered as potential threats to the survival of Australian orchids. Unfortunately, because it is not possible to predict how the variables listed above will change in various scenarios of climate change we could not incorporate these factors in our analyses.

To summarize, the analyses revealed that climate change will adversely affect sexually deceptive orchids, but at the same time the pollinator of *C*. *leptochila* will benefit from global warming. In the worst-case scenario this orchid will lose 75% of its currently suitable niches, but even in its very limited geographical range the availability of the pollen vector will be higher than currently. The ecological interactions and climatic variables that could not be modelled using available data can be potential additional threats to the orchid studied.

## Supporting information

S1 Annex Records of *Cryptostylis leptochila* and *Lissopimpla excelsa* georeferenced with a minimum precision of 1 km. (XLSX)

S2 Annex Records of *Cryptostylis leptochila* and *Lissopimpla excelsa* included in the ENM. (XLSX)

S1 Table List of variables used in the modelling. (DOCX)

S2 Table Results of the statistical evaluation of the models. (DOCX)

S3 Table Calendar of the occurrence the flowering of *C*. *leptochila* and activity of *L*. *excelsa* in four periods of time (1951–1980, 1981–2000, 2001–2010, 2011–2020) compiled based on public databases. (DOCX)
