# Supplementary material for: The effect of global warming on the Australian endemic orchid Cryptostylis leptochila and its pollinator
Source: PLoS One. 2023 Jan 30;18(1):e0280922. doi: 10.1371/journal.pone.0280922 (PMC9886262; doi:10.1371/journal.pone.0280922)
Supplement: S1 Table — (DOCX) [file pone.0280922.s003.docx]

S1 Table. List of variables used in the modeling.

| **Variable group** | **Variable code** | **Description** |
| --- | --- | --- |
| bioclims | bio1 | Annual Mean Temperature |
|  | bio2 | Mean Diurnal Range = Mean of monthly (max temp − min temp) |
|  | bio3 | Isothermality (bio2/bio7) * 100 |
|  | bio4 | Temperature Seasonality (standard deviation ×100) |
|  | bio5 | Max Temperature of Warmest Month |
|  | bio6 | Min Temperature of Coldest Month |
|  | bio8 | Mean Temperature of Wettest Quarter |
|  | bio9 | Mean Temperature of Driest Quarter |
|  | bio12 | Annual Precipitation |
|  | bio13 | Precipitation of Wettest Month |
|  | bio14 | Precipitation of Driest Month |
|  | bio15 | Precipitation Seasonality (Coefficient of Variation) |
|  | bio18 | Precipitation of Warmest Quarter |
|  | bio19 | Precipitation of Coldest Quarter |
| Soil class (Soil_type) | 0 | Acrisols |
|  | 1 | Albeluvisols |
|  | 2 | Alisols |
|  | 3 | Andosols |
|  | 4 | Arenosols |
|  | 5 | Calcisols |
|  | 6 | Cambisols |
|  | 7 | Chernozems |
|  | 8 | Cryosols |
|  | 9 | Durisols |
|  | 10 | Ferralsols |
|  | 11 | Fluvisols |
|  | 12 | Gleysols |
|  | 13 | Gypsisols |
|  | 14 | Histosols |
|  | 15 | Kastanozems |
|  | 16 | Leptosols |
|  | 17 | Lixisols |
|  | 18 | Luvisols |
|  | 19 | Nitisols |
|  | 20 | Phaeozems |
|  | 21 | Planosols |
|  | 22 | Plinthosols |
|  | 23 | Podzols |
|  | 24 | Regosols |
|  | 25 | Solonchaks |
|  | 26 | Solonetz |
|  | 27 | Stagnosols |
|  | 28 | Umbrisols |
|  | 29 | Vertisols |
| Soil properties | Clay05 | Clay content |
|  | Ph05 | Soil pH in H_2_O |
|  | Sand05 | Sand content |
|  | SOC05 | Soil organic carbon content |
|  | Nitrogen05 | Nitrogen content |
| Land cover (GMLC) | 1 | Broadleaf Evergreen Forest |
|  | 2 | Broadleaf Deciduous Forest |
|  | 3 | Needleleaf Evergreen Forest |
|  | 4 | Needleleaf Deciduous Forest |
|  | 5 | Mixed Forest |
|  | 6 | Tree Open |
|  | 7 | Shrub |
|  | 8 | Herbaceous |
|  | 9 | Herbaceous with Sparse Tree/Shrub |
|  | 10 | Sparse vegetation |
|  | 11 | Cropland |
|  | 12 | Paddy field |
|  | 13 | Cropland / Other Vegetation Mosaic |
|  | 14 | Mangrove |
|  | 15 | Wetland |
|  | 16 | Bare area,consolidated (gravel,rock) |
|  | 17 | Bare area,unconsolidated (sand) |
|  | 18 | Urban |
|  | 19 | Snow / Ice |
|  | 20 | Water bodies |
