# Supplementary material for: The effect of global warming on the Australian endemic orchid Cryptostylis leptochila and its pollinator
Source: PLoS One. 2023 Jan 30;18(1):e0280922. doi: 10.1371/journal.pone.0280922 (PMC9886262; doi:10.1371/journal.pone.0280922)
Supplement: S2 Table — (DOCX) [file pone.0280922.s004.docx]

S2 Table. Results of models evaluation statistics.

|  | AUC | | TSS | |
| --- | --- | --- | --- | --- |
|  | *C. leptochila* | *L. excelsa* | *C. leptochila* | *L. excelsa* |
| present time | 0.990 (0.000) | 0.965 (0.005) | 0.964 | 0.780 |
| SSP 1-2.6 | 0.988 (0.001) | 0.967 (0.006) | 0.964 | 0.794 |
| SSP 2-4.5 | 0.990 (0.000) | 0.966 (0.006) | 0.976 | 0.792 |
| SSP 3-7.0 | 0.990 (0.000) | 0.967 (0.005) | 0.973 | 0.860 |
| SSP 5-8.5 | 0.990 (0.000) | 0.968 (0.005) | 0.966 | 0.807 |
