# Supplementary material for: The effect of global warming on the Australian endemic orchid Cryptostylis leptochila and its pollinator
Source: PLoS One. 2023 Jan 30;18(1):e0280922. doi: 10.1371/journal.pone.0280922 (PMC9886262; doi:10.1371/journal.pone.0280922)
Supplement: S3 Table — (DOCX) [file pone.0280922.s005.docx]

S3 Table. Calendarial of flowering time of *C. leptochila* and activity of *L. excelsa* in four time periods (1951-1980, 1981-2000, 2001-2010, 2011-2020) compiled based on public databases.

| **Species** | **Time period** | **Month** | | | | | | | | | | | |
| --- | --- | --- | --- | --- | --- | --- | --- | --- | --- | --- | --- | --- | --- |
|  |  | I | II | III | IV | V | VI | VII | VIII | IX | X | XI | VII |
| *C. leptochila* | 1951-1980 | **x** | **x** | **x** | **x** | **x** | **x** |  | **x** | **x** | **x** | **x** | **x** |
| *L. excelsa* | 1951-1980 |  |  | x | x |  |  |  |  |  | x |  | x |
| *C. leptochila* | 1981-2000 | **x** | **x** | **x** | **x** | **x** | **x** |  | **x** | **x** | **x** | **x** | **x** |
| *L. excelsa* | 1981-2000 | x | x |  | x | x |  |  |  | x | x | x | x |
| *C. leptochila* | 2001-2010 | **x** | **x** | **x** | **x** | **x** | **x** |  | **x** | **x** | **x** | **x** | **x** |
| *L. excelsa* | 2001-2010 |  | x |  | x |  |  |  | x |  | x | x | x |
| *C. leptochila* | 2011-2020 | **x** | **x** | **x** |  | **x** | **x** |  | **x** | **x** | **x** | **x** | **x** |
| *L. excelsa* | 2011-2020 | x | x | x | x | x | x | x | x | x | x | x | x |
